# Hypoxic Conditioning: A Potential Perioperative Strategy to Reduce Abdominal Aortic Occlusion-Related Injury in Mouse Proximal and Distal Organs

**DOI:** 10.14336/AD.2024.0121

**Published:** 2024-01-24

**Authors:** Changhong Ren, Ning Li, Jun Xu, Yong Yang, Linhui Qin, Linpei Jia, Xian Wang, Shuangfeng Huang, Kunlin Jin, Fengyong Liu, Xunming Ji, Sijie Li

**Affiliations:** ^1^Beijing Key Laboratory of Hypoxia Conditioning Translational Medicine, Xuanwu Hospital, Capital Medical University, Center of Stroke, Beijing Institute of Brain Disorder, Capital Medical University, Beijing, China.; ^2^School of Chinese Medicine, Beijing University of Chines Medicine, Beijing, China; ^3^Department of Nephrology, Xuanwu Hospital, Capital Medical University, Beijing, China.; ^4^Department of Pharmacology & Neuroscience, University of North Texas Health Science Center, Fort Worth, TX 76107, USA.; ^5^Department of Interventional Radiology, Senior Department of Oncology, Fifth Medical Center of PLA General Hospital, Beijing, China

**Keywords:** abdominal aortic occlusion, hypoxic conditioning, inflammation, apoptosis

## Abstract

This study aimed to investigate the impact of abdominal aortic occlusion (AAO)- induced injury on the kidney, lower limb muscles, heart, and brain in mice, and the potential protective effects of hypoxic postconditioning (HyC). The experimental design employed an abdominal aortic occlusion (AAO) model, and involved three groups of mice: sham, AAO, and AAO+HyC. Ten minutes after the AAO model, mice were subjected to hypoxic treatment lowering oxygen concentration to 5% within 45 minutes, and then returned to a normal oxygen environment. Hematoxylin- eosin (HE) stain was used for Histopathological examinations, and Quantibody Mouse Array was used for detecting apoptosis and inflammation-related protein expression. Histopathological examinations showed that HyC mitigated pathological damage to proximal organs (kidneys and lower limb muscles), distal organs (heart and brain), and reduced inflammatory cell infiltration. Expression of apoptosis- and inflammation-related proteins in brain and heart tissues were also evaluated. HyC significantly increased cellular inhibitor of apoptosis 2 (cIAP2) in the brain and Bcl-2 and insulin-like growth factor 2 (IGF-2) in the heart. Additionally, HyC regulated the expression of several inflammation-related factors in both brain and heart tissues. Although further investigation is needed, particularly in human subjects, this study highlights the potential of HyC as a promising therapeutic strategy for reducing AAO-associated organ damage.

## INTRODUCTION

Abdominal aortic occlusion (AAO), also referred to as aortic clamping, is a surgical procedure that involves temporary or permanent occlusion of the abdominal aorta [1]. This procedure has various clinical applications such as controlling bleeding from the abdominal organs, abdominal aortic aneurysm repair, organ transplantation and providing a bloodless field for surgical procedures [2].

However, AAO also carries a significant risk of ischemia and ischemia-reperfusion (I/R) injury, which can result in tissue damage and cell death. Ischemia, the interruption of blood supply to an organ or tissue, and subsequent reperfusion after aortic clamping, can exacerbate tissue damage through the release of free radicals, inflammatory cytokines, and other toxic substances [3]. I/R injury can impact multiple organs, ranging from proximal ones (e.g., liver, kidneys, lower limb muscles, intestines) to distal ones (e.g., lungs, heart, brain) [4, 5]. These complications can lead to postoperative issues such as organ dysfunction, sepsis, and even death [6].

To minimize the risk of ischemia-reperfusion injury in the perioperative period, various techniques have been developed [7, 8], including selective organ perfusion, pharmacological interventions, and the use of protective agents such as antioxidants. The development of new therapeutic interventions and strategies are critical areas of research to mitigate the tissue damage and cell death caused by AAO and I/R injury. This will improve patient outcomes and reduce the burden of chronic disease associated with these conditions [4].

Hypoxic conditioning (HyC), a training method aimed at improving performance by exposing the body to reduced oxygen levels, has been used by athletes for years. This type of training has been used by athletes for years as a means of improving their performance, endurance, and recovery time [9]. In recent years, HyC has gained attention for its potential health benefits beyond athletic performance. Exposure to low oxygen levels has been shown to stimulate various physiological responses in the body, including the production of new blood vessels, increased oxygen delivery to tissues, and improved cellular energy metabolism [10]. These effects have led to interest in the potential therapeutic applications of HyC for conditions such as cardiovascular disease, stroke, and neurological disorders [11, 12]. Studies showed that HyC can stimulate the production of antioxidants, reduce oxidative stress and increase the expression of anti-inflammatory cytokines, such as interleukin-10, which can reduce the inflammatory response to I/R injury [10, 12].

Several studies demonstrate HyC's effectiveness in reducing postoperative myocardial injury/infarction incidents [13], cerebral ischemia [14], and renal impairment in preclinical studies [15], the protective effect of HyC on proximal organs (such as the kidney and lower limb muscles) and distal organs (such as the heart and brain) caused by AAO is still unclear. Additionally, the underlying molecular mechanisms involved in these changes have not been fully explored. To investigate these effects, we employed a mouse model of AAO, examining pathological changes in both proximal and distal organs. Additionally, we explored the potential molecular mechanisms by using both quantitative and semi-quantitative protein arrays to measure inflammatory factors and apoptosis-related proteins, respectively. This study sheds light on the complex interplay between abdominal aortic ligation and the broader functioning of the body, providing valuable insights for clinicians and researchers alike.

**Figure 1.**
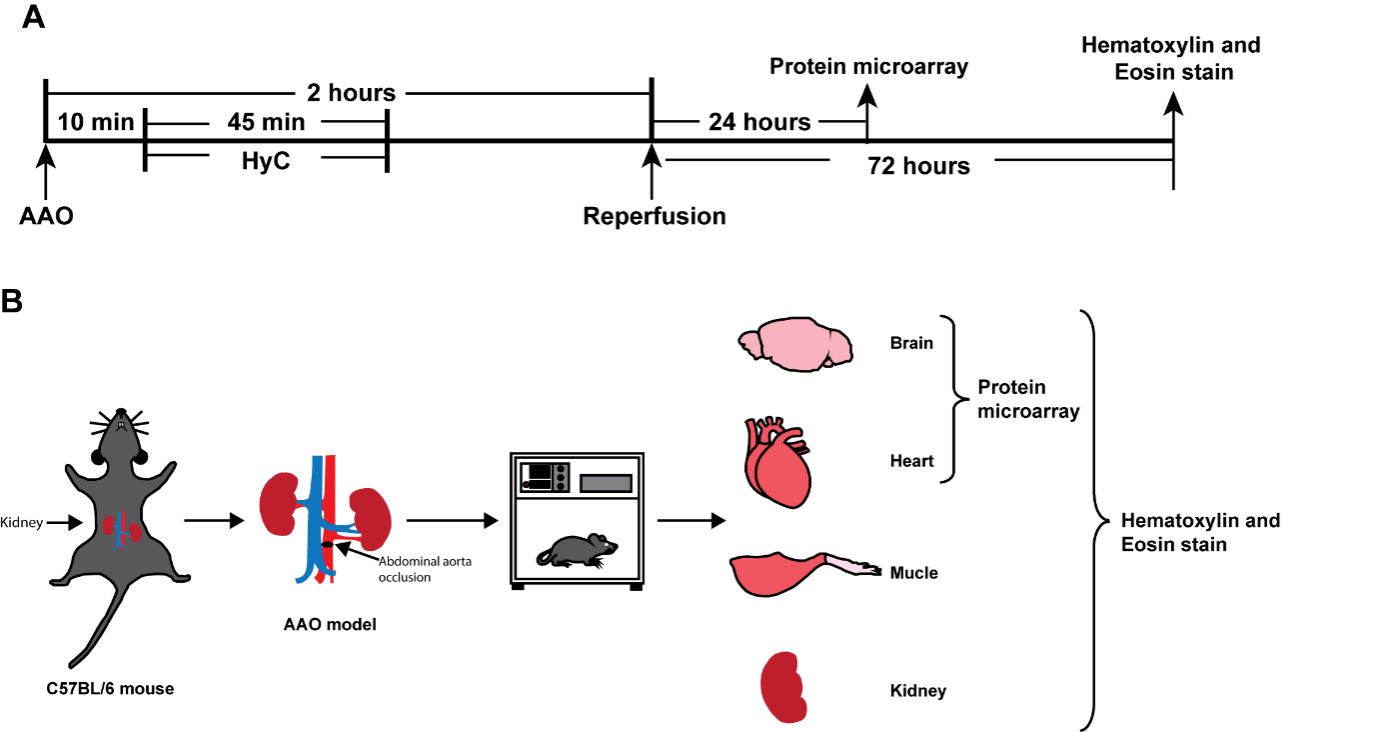
**Schematic presentation of the experimental design used in this study**. Male C57BL/6 mice were randomly assigned to either the sham group or the AAO group. In the sham group, only a laparotomy was performed. In the AAO group, the abdominal aorta under the left renal 0.5 cm was occluded with sutures for 2 hours. **(A)** The timeline of the experimental process. **(B)** Diagram of AAO site and diagram of HyC and extracted tissues. AAO, abdominal aorta occlusion.

## MATERIALS AND METHODS

### Animals

The study utilized eight-week-old male C57BL/6 mice weighing 20-23 g, which were purchased from Vital River Laboratory Animal Technology Co. Ltd. The mice were housed in a temperature-controlled room maintained at 23 ± 1 ?, with a 12-hour light-dark cycle and provided with ad libitum access to food and water [1]. All animal experiments in this study were conducted in accordance with the guidelines outlined in the Guide for the Care and Use of Laboratory Animals and approved by the Institutional Animal Care and Use Committee of Capital Medical University on October 14th, 2021 (approval number: 2021/255).

### Abdominal aortic ischemia/reperfusion injury model

In this investigation, the AAO model was employed, as previous descripted [16], to examine the impact of I/R injury on the kidney, lower limb muscles, heart and brain. We randomly divided the mice into three groups: sham, AAO, and AAO+HyC. The mice were anesthetized with 1.5% enflurane, 30% O_2_, and 68.5% N_2_O, and underwent a midline incision after shaving and disinfection. In the sham group, a laparotomy was performed, while in the AAO group, the abdominal aorta was occluded under the left renal for 2 h using sutures. The body temperature of the mice was regulated throughout the operation using a temperature-controlling pad (Harvard Apparatus, MA, USA). After blood sampling, the animals were sacrificed, and neurologic and cardiac tissue samples were obtained from all mice. The flow chart of experimental design is shown in Figure 1.

### Hypoxic postconditioning (HyC) treatment

After 10 min of AAO model, mice were placed in a hypoxic chamber, and hypoxic treatment was initiated to reduce the oxygen concentration to 5% within 45 min. After 45 min of treatment, the mice were returned to a normal oxygen environment[17].

### Biochemical analyses

Blood was obtained at 24h after reperfusion, and serum was isolated at 3000 rpm/mi for 10 min. Serum levels of total bilirubin (TBIL), alkaline phosphatase (ALP), blood urea nitrogen (BUN), creatinine (Cr) and lactic dehydrogenase (LDH) were detected by an automatic biochemical analyzer (Mindray Medical International Co. Ltd., Shenzhen, Guangdong, China), according to the specifications for instrument operation. Serum level of cardiac troponin I (cTnI) was detected using an enzyme-linked immunosorbent assays kit (Uscn Life Science, Inc., Wuhan, China) according to the manufacturer’s instructions.

### Hematoxylin- eosin (HE) stain and Sirius red staining

The mouse is anesthetized and transcardially perfused with saline followed by a 4% paraformaldehyde fixative solution at 72 h after reperfusion. The tissue of brain, heart, kidney, and muscle tissues are then removed and post-fixed in the same fixative solution. Next, the fixed tissue is embedded in paraffin wax and sectioned into thin slices (4.5 μm). To perform HE staining on paraffin-embedded sections of mice, the tissue sections were deparaffinized with xylene, and then rehydrated with graded ethanol solutions. Hematoxylin was used to stain nuclei blue-purple. An acidic solution was used to control staining intensity. Eosin was used to stain cytoplasm and extracellular matrix pink (G1120, Solarbio Science & Technology Co., Ltd, Beijing, China). The sections were dehydrated with graded ethanol solutions (70%, 95%, and 100%), and then cleared with xylene. Finally, a mounting medium was used to mount them on glass slides [5]. After HE staining, the tissue sections were examined under a light microscope to visualize the different cellular and tissue structures. The nuclei will appear blue-purple, while the cytoplasm and extracellular matrix will appear pink. Calculated the edematous renal tubules in renal cortex of mice, the infiltration area of inflammatory cells in lower limb muscle of mice, the area of eosinophilic myocardial fibers in heart in low-power fields of sections. Damaged neuronal cells and swollen neurons in brain was quantified in high-power fields of sections. For each mouse, we selected three visual fields of the same part for statistics.

To visualize collagen fibers in heat-deparaffinized tissue sections, Sirius red staining was employed. The sections were incubated in the Sirius red solution at room temperature for 1 h (G1472, Solarbio Science & Technology Co., Ltd, Beijing, China). To remove excess dye, the tissue sections were rinsed in a sodium chloride solution for 2 min. Subsequently, the sections were dehydrated by rinsing them in a series of ethanol solutions (70%, 95%, and 100%). In order to increase the birefringence of the collagen fibers, the tissue sections underwent a treatment with acidified water consisting of 0.5% glacial acetic acid in distilled water for a duration of 2 min. Subsequently, the treated tissue sections were mounted using a suitable medium and covered with a coverslip. The tissue slice panoramic scanning system (Pannoramic MIDI, 3DHistech Company, Hungary) was used to take photos. Three low-power fields of each mouse were examined. Tissues damage was quantified using previously published scoring criteria [4]. The intensity of the staining was scored on a scale from 0 to 3 (0= no staining; 1= weak staining; 2= moderate staining; 3= strong staining).

### Fluoro-Jade C staining (FJC)

FJC staining was performed according to the manufacturer’s protocol (Biosensis, SA, Australia). Paraffin-embedded brain sections were deparaffinized in xylene for 5 minutes, repeated three times, and then rehydrated through a series of ethanol washes. The slides were then incubated in a potassium permanganate solution for 10 minutes and subsequently rinsed in distilled water for 2 minutes. Following this, the slides were stained with Fluoro-Jade C solution containing DAPI for 10 minutes and washed in distilled water for 1 minute, repeated three times. The slides were dried at a temperature between 50-60 °C for a minimum of 5 minutes. Once dry, the slides were cleared in xylene for 5 minutes and then mounted with DPX mounting medium. Fluoro-Jade C-positive cells were quantified using ImageJ software.

### Protein expression assay

Protein expression was evaluated in brain and heart samples at 24 h after reperfusion using the Quantibody Mouse Inflammation Array 1 and Mouse Apoptosis Array C1 (Raybiotech, Norcross, GA). Samples were minced and incubated in the respective sample diluent buffer for 1 h at 37 °C before being homogenized. The homogenate was centrifuged at 13,000 g for 5 min to remove debris and insoluble material. Total protein content was determined using the bicinchoninic acid method, and protein extracts were prepared at a concentration of 200 μg/ml. The Quantibody Mouse Inflammation Array 1 was used to quantify 40 cytokines, while Mouse Apoptosis Array C1 was used to semi-quantitatively measure 38 apoptosis-related proteins. The binding of each protein on the membrane was revealed by autoradiography and quantified using the Protein Array Analyzer for ImageJ program developed for ImageJ software (http://rsb.info.nih.gov/ij/macros/toolsets/Protein%20Array%20Analyzer.txt). Each assay was performed in duplicate from three mice per experimental group for each day tested.

### Western blot

Mice were anesthetized and perfused with normal saline. Then brains and hearts were obtained. After protein extraction, proteins were separated in SDS-PAGE gels by electrophoresis and transferred to a PVDF membrane. Primary antibodies incubation at 4 °C overnight. Primary antibodies against HIF-1α (1:1000; Cell Signaling Technology, Boston, Mass, USA) and β-actin (1:2000, Zhongshan Golgen Bridge, Beijing, China). Secondary antibodies with an HRP (1:2000, Zhongshan Golgen Bridge, Beijing, China) were incubated at room temperature for 2 h. Protein bands were detected using an enhanced chemiluminescence kit (Thermo Fisher Scientific, Waltham, Mass, USA).

### Statistical analysis

All values were presented as mean ± SEM. The sample size and accompanying P values are stated in each figure. All experiments were conducted in a randomized fashion, and the researcher performing the experiments was blinded to the group and treatment assignments. The normality of the data was assessed using the Shapiro-Wilks test. For data sets that exhibited a normal distribution, greater or equal to three groups were analyzed using One-way ANOVA analysis followed by Tukey *post-hoc* test. For data that did not pass the normality test (or n<6), Kruskal-Wallis test with Dunn's *post-test* (for more than two groups) was utilized. Statistical analysis was performed using GraphPad Prism version 6.0 (GraphPad Software, San Diego, CA, USA). A significance level of *p*<0.05 was considered statistically significant to draw conclusions.

**Table 1:** Serum levels of function injury markers.

Variable	group		
	sham	AAO	AAO+HyC
TBIL (umol/L)	4.39±0.23	3.96±0.37	4.23±0.33
ALP (U/L)	257±19.81	265.4±20.05	259.2±21.16
BUN (mmol/L)	3.89±0.28	9.21±1.5^**^	4.9±0.8^&^
Cr (umol/L)	37.49±4.36	59.96±6.58^*^	41.16±4.03^&^
LDH (U/L)	1472±151.79	2098±240.41	1559.6±202.89
cTnI (nmol/L)	1.59±0.37	2.93±0.32^*^	1.65±0.36^&^

** *vs.* sham, P<0.01; & *vs.* AAO, P<0.05; * *vs.* sham, P<0.05

## RESULTS

### HyC mitigated the functional damage inflicted by AAO

To investigate the functional changes of various organs following HyC treatment, we analyzed the biomarkers in the blood that indicate organ dysfunction. Table 1 showed that there were no significant differences in TBIL and ALP levels between the sham group and the AAO group, or between the AAO+HyC group and the AAO group. This suggested that there was no liver functional damage and that HyC did not affect liver function. However, the levels of BUN and Cr were significantly increased in the AAO group compared to the sham group (P<0.01 and P<0.05, respectively). HyC administration resulted in a decrease in these levels (P<0.05), indicating that the renal function of mice with AAO was impaired and that HyC alleviated the renal functional damage caused by AAO. Similarly, there was a significant increase in cTnI levels in the AAO group compared to the sham group (P<0.05). However, HyC administration resulted in a decrease in cTnI levels in the blood (P<0.05), suggesting that AAO induced myocardial injury, which was mitigated by HyC treatment.

**Figure 2.**
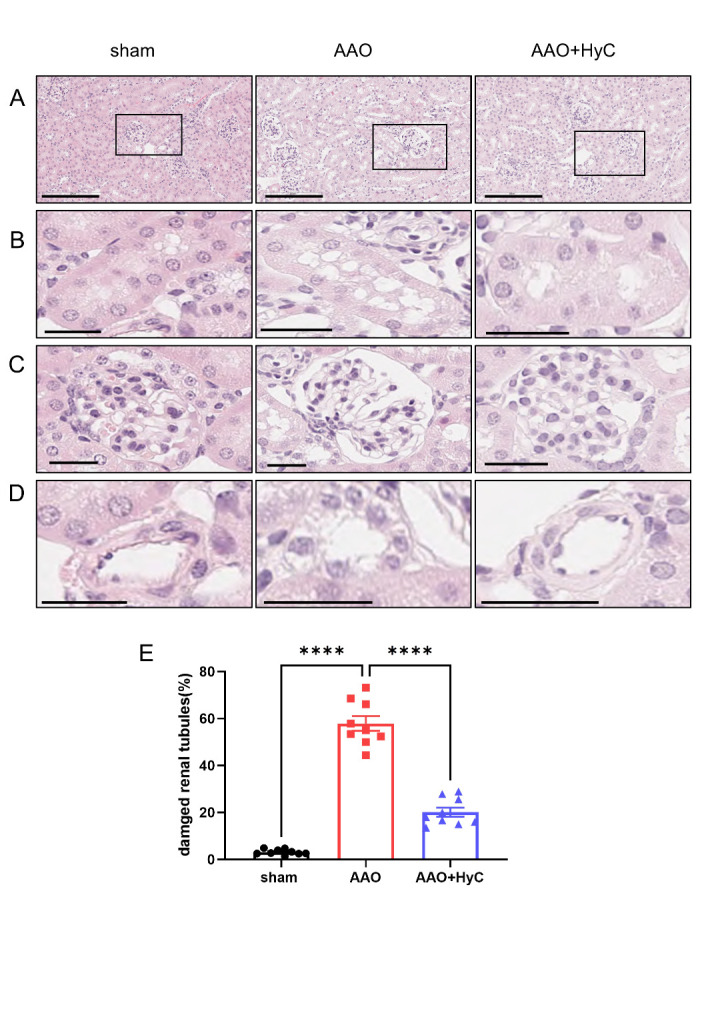
**HyC mitigated the pathological damage inflicted by AAO on kidneys. (A)** Representative HE staining of the renal cortex. Scale bar =200μm. **(B)** Representative image of renal tubule of enlarged view of the black box area in the Figure A. Scale bar =20μm. **(C)** Representative image of glomeruli of enlarged view of the black box area in the figure A. Scale bar =20μm. **(D)** Representative image of vessel of enlarged view of the black box area in the figure A. Scale bar =20μm. **(E)** The bar graph showed the percentage of damaged damaged renal tubules in each group. One-way ANOVA with Tukey’s honestly post hoc test was used. N = 3 mice per group, with 3 fields of view analyzed per mouse. ****P<0.0001. Bar graphs: mean ± SEM.

### HyC mitigated the pathological damage inflicted by AAO on proximal organs, kidneys, and lower limb muscle

To investigate the effect of perioperative strategy of HyC on an organ-specifc manner, we proceeded with histopathological examination of kidney and lower limb muscle. HE staining showed that renal tubule glomeruli and blood vessels in the renal cortex of mice in the sham group had regular shape, clear boundaries, no edema phenomenon, and uniform cytoplasmic and nuclear staining. In the AAO group, we observed renal tubule edema, more vacuoles, vascular swelling, morphology destruction, glomeruli swelling, blurred boundaries, dark nucleus staining, and enlarged renal cortical space. However, the group treated with HyC demonstrated a reduction in the pathological damage (Fig. 2A-D). The percentage of edematous renal tubules was increased in the AAO group compared to the sham group (Fig. 2E) (*P*<0.0001), HyC decreased it (Fig. 2E) (*P*<0.0001).

**Figure 3.**
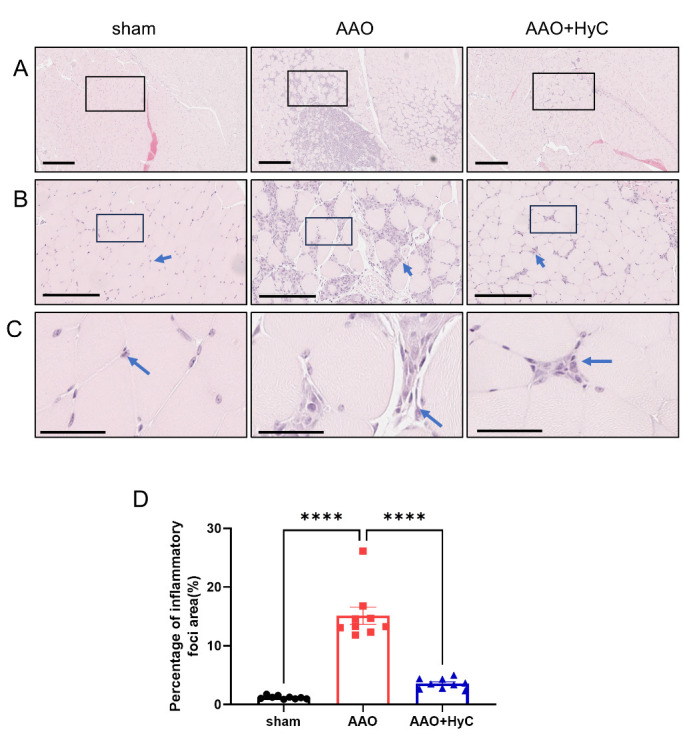
**HyC mitigated the pathological damage inflicted by AAO on lower limb muscle. (A)** HE staining of mouse quadriceps muscle. Scale bar =400μm; **(B)** An enlarged view of the black box area in the Figure A. Blue arrow indicated inflammatory foci. Scale bar =200μm; **(C)** An enlarged view of the black box area in the Figure B. Blue arrow indicated inflammatory foci. Scale bar =50μm. **(D)** The bar graph showed the percentage of inflammatory foci area in each group. One-way ANOVA with Tukey’s honestly post hoc test was used. N = 3 mice per group, with 3 fields of view analyzed per mouse., * p<0.05, ** p<0.01. **** P<0.0001. Bar graphs: mean ± SEM.

The HE staining of the quadriceps muscle of mice showed that the skeletal muscle cells of mice in the sham group were neatly arranged, with smaller cell gaps and less inflammatory cell infiltration. In the AAO group, there were a large number of inflammatory cells infiltrating, and the cell space increased. However, the inflammatory infiltration of lower limb muscles of mice in the AAO+HyC group decreased, and the cell space became smaller (Fig. 3). The area with inflammatory cell infiltration was significantly increased in the AAO group compared with the sham group (Fig. 3C) (*P*<0.0001). The AAO+HyC group exhibited a significant decrease in the area compared with the AAO group *(P*<0.0001).

### HyC mitigated the pathological damage inflicted by AAO on distal organs, heart and brain

**Figure 4.**
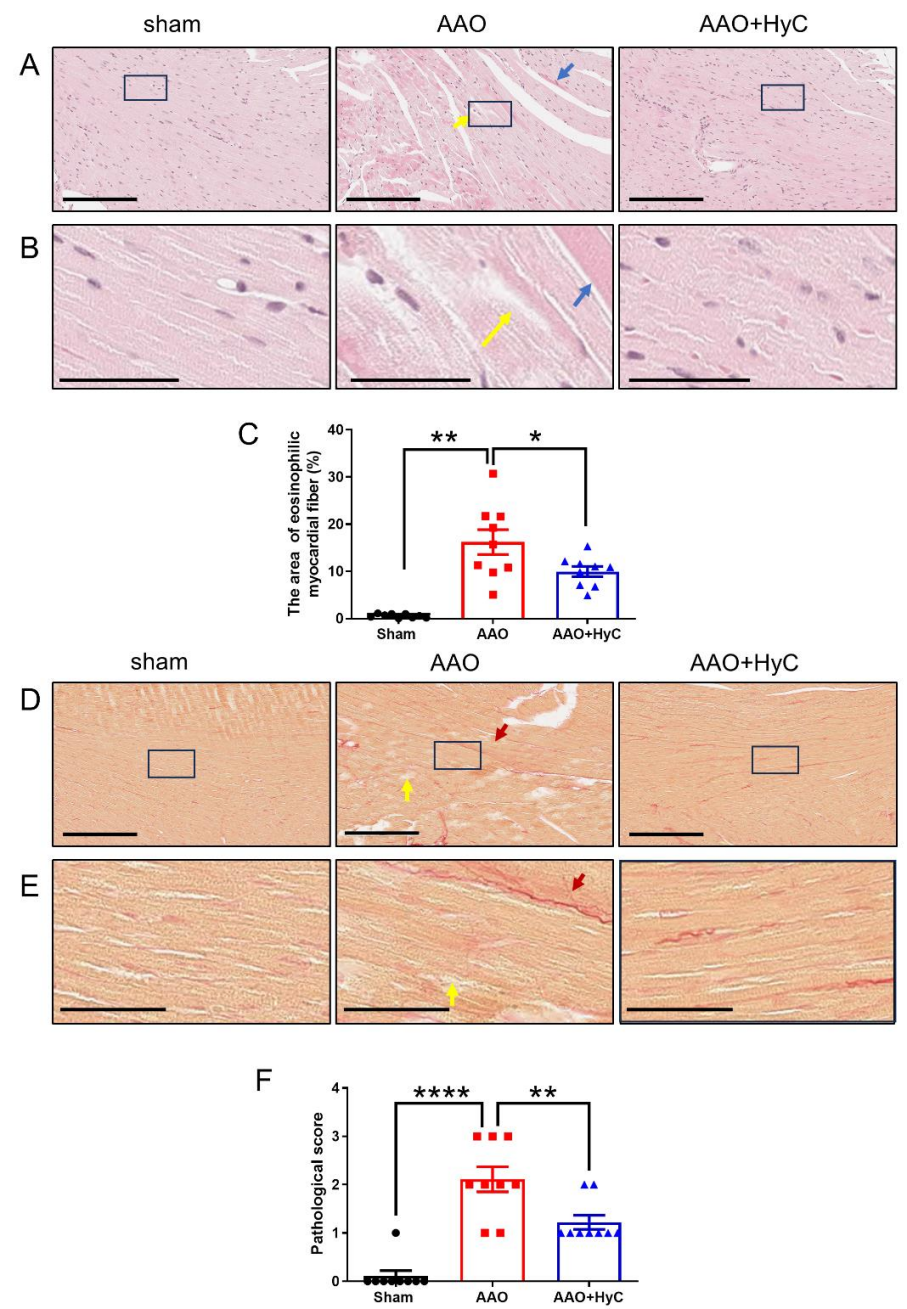
**HyC mitigated the pathological damage inflicted by AAO on the heart. (A)** HE staining of the right anterior wall of the mouse left ventricle. The yellow arrow pointed to morphologically destroyed myocardial fiber. The blue arrow pointed to eosinophilic myocardial fibers. Scale bar =200μm. **(B)** An enlarged view of the black box area in figure A. Scale bar =50μm. **(C)** The bar graph showed the area percentage of eosinophilic myocardial fibers. One-way ANOVA with Tukey’s honestly post hoc test was used. N = 3 mice per group, with 3 fields of view analyzed per mouse. *P<0.05, **P<0.01. Bar graphs mean ± SEM, n=10/group. **(D)** Representations of Sirius red staining of the right anterior wall of the left ventricle in mice. The red arrow pointed to deposition of collagen fibers and the yellow arrow pointed to morphologically destroyed cardiomyocytes. Scale bar =200μm. **(E)** An enlarged view of the black box area in the Figure D. Scale bar =50μm. **(F)** The bar graph showedthe pathologicall score of each group. One-way ANOVA with Tukey’s honestly post hoc test was used. N = 3 mice per group, with 3 fields of view analyzed per mouse. *p<0.01, ****P<0.0001. Bar graphs: mean ± SEM.

HE staining of the right anterior wall of the left ventricle showed that the myocardial cells of mice in the sham group were neatly arranged, continuous and compact, with uniform cytoplasm and no edema. In the AAO group, the myocardial cells were disordered, blurred and swollen, with morphological destruction, increased larger tissue space. AAO+HyC group improved cell arrangement and swelling, and decreased tissue space (Fig. 4A, B). A great deal of eosinophilic myocardial fibers can be observed in AAO group (The blue arrow in Fig. 4B is shown). The area of eosinophilic myocardial fibers was significantly increased in the AAO group compared with the sham group (Fig. 4C) (*P*<0.01). The AAO+HyC group exhibited a significant decrease in the area compared with the AAO group (*P*<0.05) (Fig. 4C).

Sirius red staining showed that the myocardial cells of mice in the sham group were arranged continuously with normal cell morphology and no obvious collagen fiber deposition in the tissue. The arrangement of myocardial cells in the AAO group was disordered, the tissue space was enlarged, and the collagen fiber deposition was increased (Fig. 4D, E). The myocardial injury of mice in AAO+HyC group was improved. Pathological score showed that HyC treatment significantly decreased the score compared with AAO groups (Fig.4F) (*p*<0.01).

**Figure 5.**
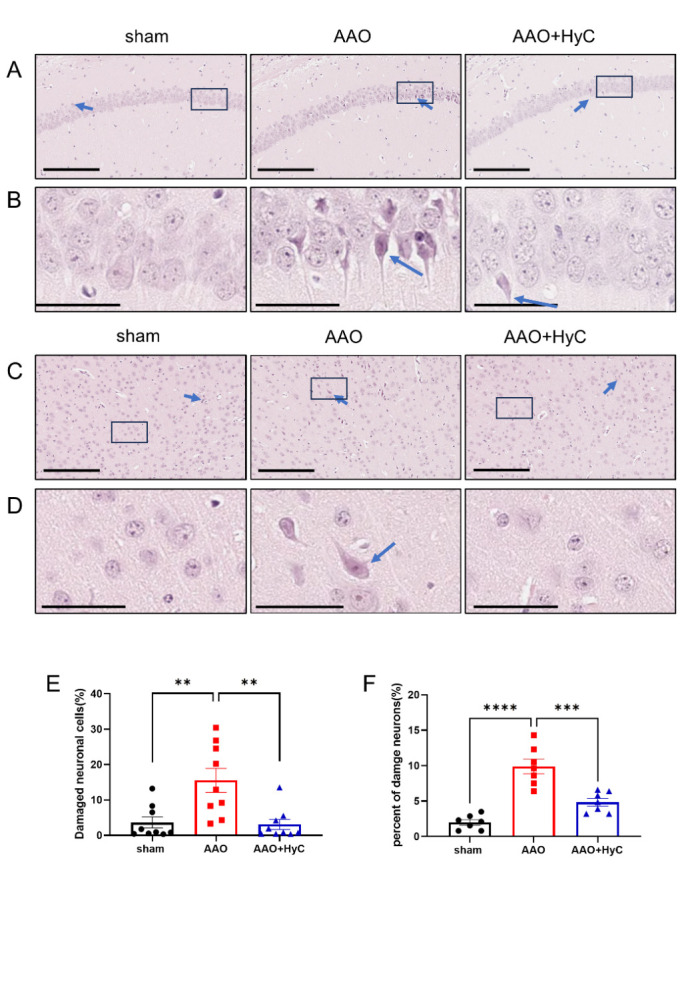
**HyC mitigated the pathological damage inflicted by AAO on the brain. (A)** showed representative images of HE staining in the CA1 region of hippocampus. The blue arrows pointed to damneurons. Scale bar = 200μm. **(B)** An enlarged view of the black box area in Figure A. Scale bar =50μm. **(C)** Representative image showed representative images of HE staining in the parietal cortex. blue arrows pointed to damaged neurons. Scale bar = 200μm. **(D)** An enlarged view of the black box area in the Figure C. Scale bar =50μm. **(E)** Showed a percentage bar graph of damaged neurons in the CA1 region of the hippocampus. **(F)** Showed a percentage bar graph of damaged neurons in the parietal cortex. One-way ANOVA with Tukey’s honestly post hoc test was used. N = 3 mice per group, with 2-3 fields of view analyzed per mouse. *p<0.05, **p<0.01. Bar graphs: mean ± SEM. ns, no significant difference.

HE staining of the hippocampus showed that in the sham group of mice, the pyramidal cells in the CA1 region were arranged neatly and tightly, with uniform staining of the cytoplasm and nucleus, and no swelling was observed (Fig. 5A, B). The neuronal morphology in the cortex was clear, and less damage was observed (Fig. 5D, E). In the AAO group, the arrangement of pyramidal cells in the CA1 region was looser, and damaged neuronal cells was observed, including chromatin aggregation in the nucleus, also known as pyknosis, and acidophilic staining of the cytoplasm (Fig. B). The number of damaged neurons in the AAO group was significantly higher than that in the sham group (*p*<0.01) (Fig. 5E). In contrast, in the AAO+HyC group, the arrangement of pyramidal cells in the CA1 region became tighter, and damaged neuronal cells decreased compared to that in the AAO group (*P*<0.01) (Fig. 5E).

**Figure 6.**
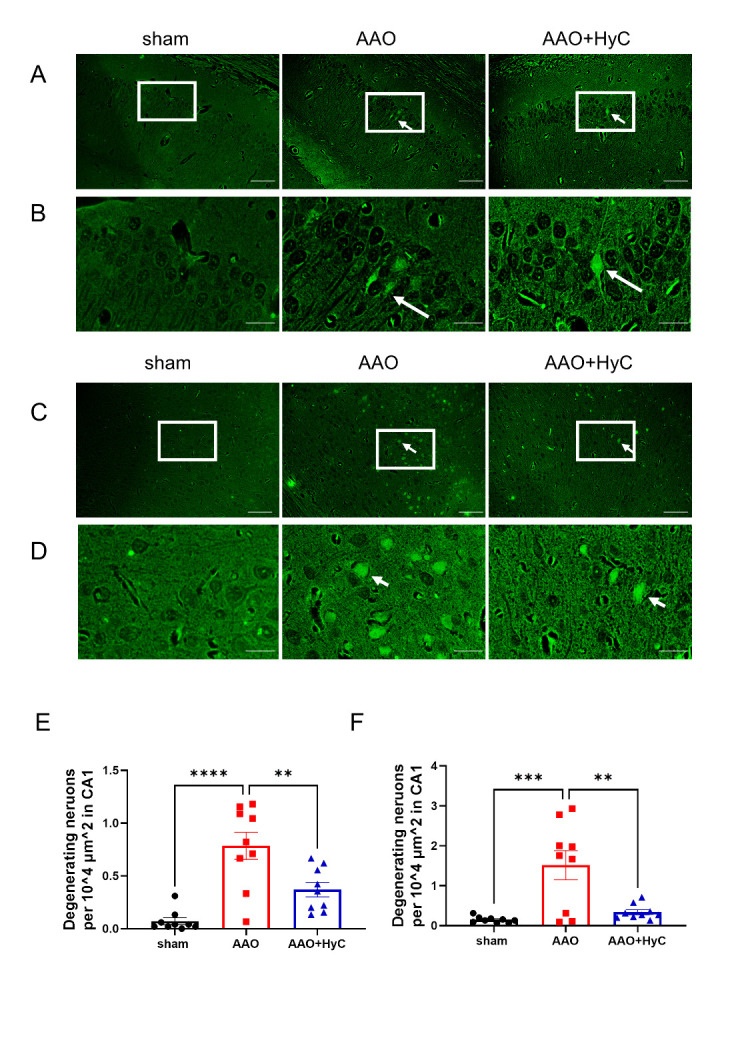
HyC reduced the degenerating neurons in the AAO brain. (A) FJC staining showing neurodegeneration of neurons in the CA1 region of hippocampus. The white arrows pointed to degenerating neurons, scale bar = 100μm. (B) An enlarged view of the white box area in the Figure A. The white arrows pointed to degenerating neurons, scale bar = 33μm. (C) Fluoro-Jade C staining showing neurodegeneration of neurons in the parietal cortex. The white arrows pointed to degenerating neurons. Scale bar = 33μm. (D) An enlarged view of the black box area in the Figure C. The white arrows pointed to degenerating neurons, scale bar = 33μm. (E) The bar graph showed the density of degenerating neurons in the CA1 region of hippocampus. (F) The bar graph showed the density of degenerating neurons in the parietal cortex. One-way ANOVA with Tukey’s honestly post hoc test was used. N = 3 mice per group, with 2 fields of view analyzed per mouse. **P<0.01, ****P<0.0001. Bar graphs: mean ± SEM.

**Figure 7.**
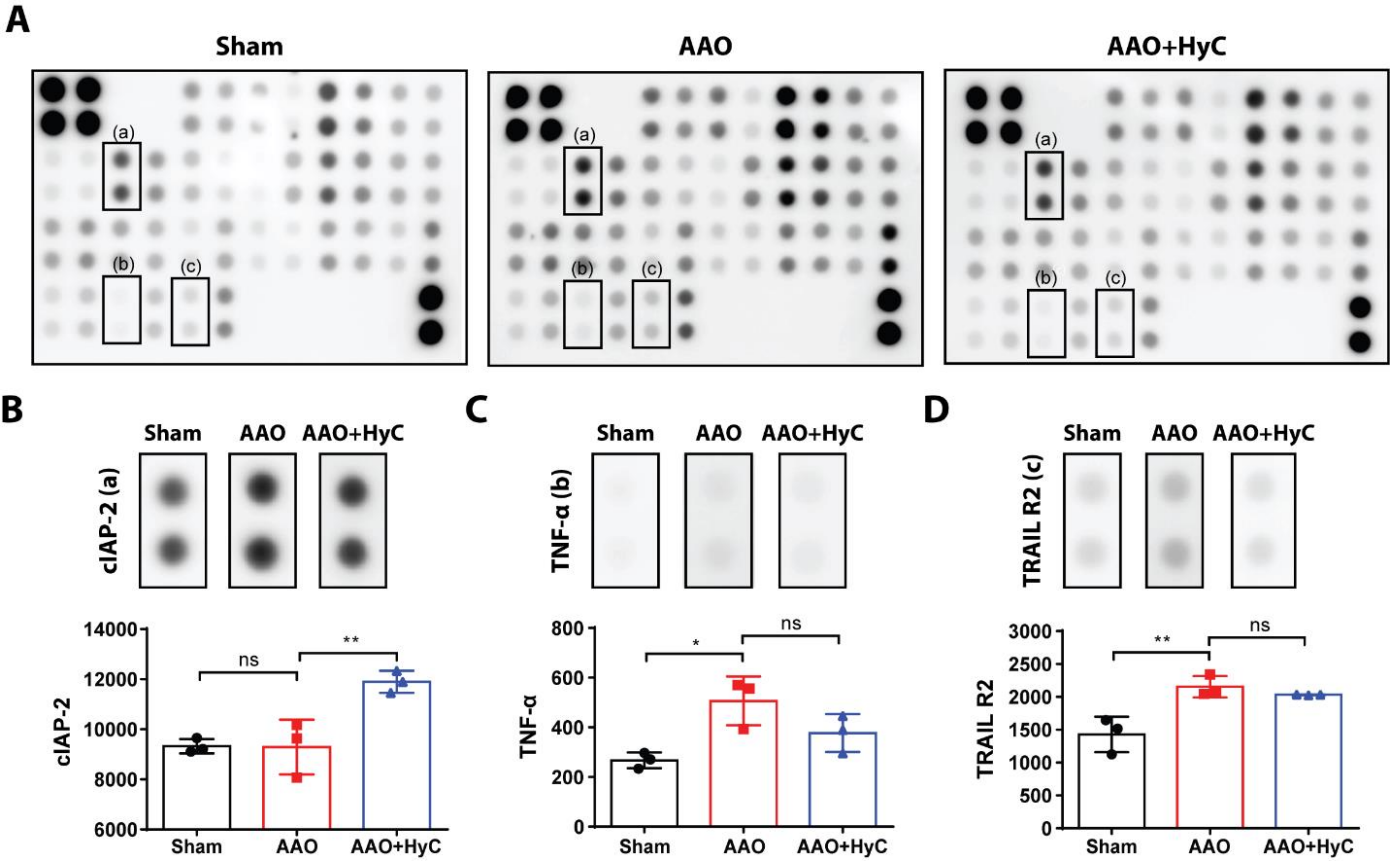
**Differential profile of apoptosis-related proteins in the brain. (A)** Representative merged images of antibody microarrays for protein detection. **(B-D)** Bar graph showed the protein semi-quantification. After examining the 38 target proteins, three exhibited marked differences in expression among the three groups in the brain. Kruskal-Wallis test with Dunn's *post hoc* test was used. *P<0.05, **P<0.01. ns, no significant difference. N=3 mice per group.

In the cortical area, the number of pyknosis neuronal cells was significantly increased in the AAO group compared with the Sham group (Fig. 5C, D, F) (*p*<0.001). In contrast, in the AAO+HyC group, the damaged neuronal cells decreased compared to that in the AAO group (Fig. 5F) (*p*<0.01). To further assess whether neuronal damage was mitigated following HyC treatment in the AAO model mice, FJC staining was employed to visualize degenerating neuronal cells. Our findings indicated that AAO resulted in a marked increase in the number of degenerate neurons within the cerebral cortex and hippocampus when compared to sham-operated mice. However, treatment with HyC led to a reduction in the number of degenerating neurons relative to the AAO group (Fig. 6).

### HyC regulated apoptosis-related proteins in brain and heart

To further investigate the underlying molecular mechanism of HyC in inhibiting apoptosis, we compared the expression levels of apoptosis-related proteins in the brain and heart among the sham, AAO, and AAO + HyC groups using an Apoptosis Antibody Array. We observed that out of the 38 target proteins, three exhibited marked differences in expression among the three groups in the brain (Fig. 7). The expression of tumor necrosis factor-α (TNF-α) and tumor necrosis factor (TNF)-related apoptosis-inducing ligand receptor 2 (TRAIL-R2) was significantly increased in the AAO group compared to the sham group (*P*<0.05) (Fig. 7). However, treatment with HyC had no effect on the two proteins. The expression of cellular inhibitor of apoptosis 2 (cIAP2) was significantly increased in the AAO+HyC group compared to the AAO group (*p*<0.01) (Fig. 7).

**Figure 8.**
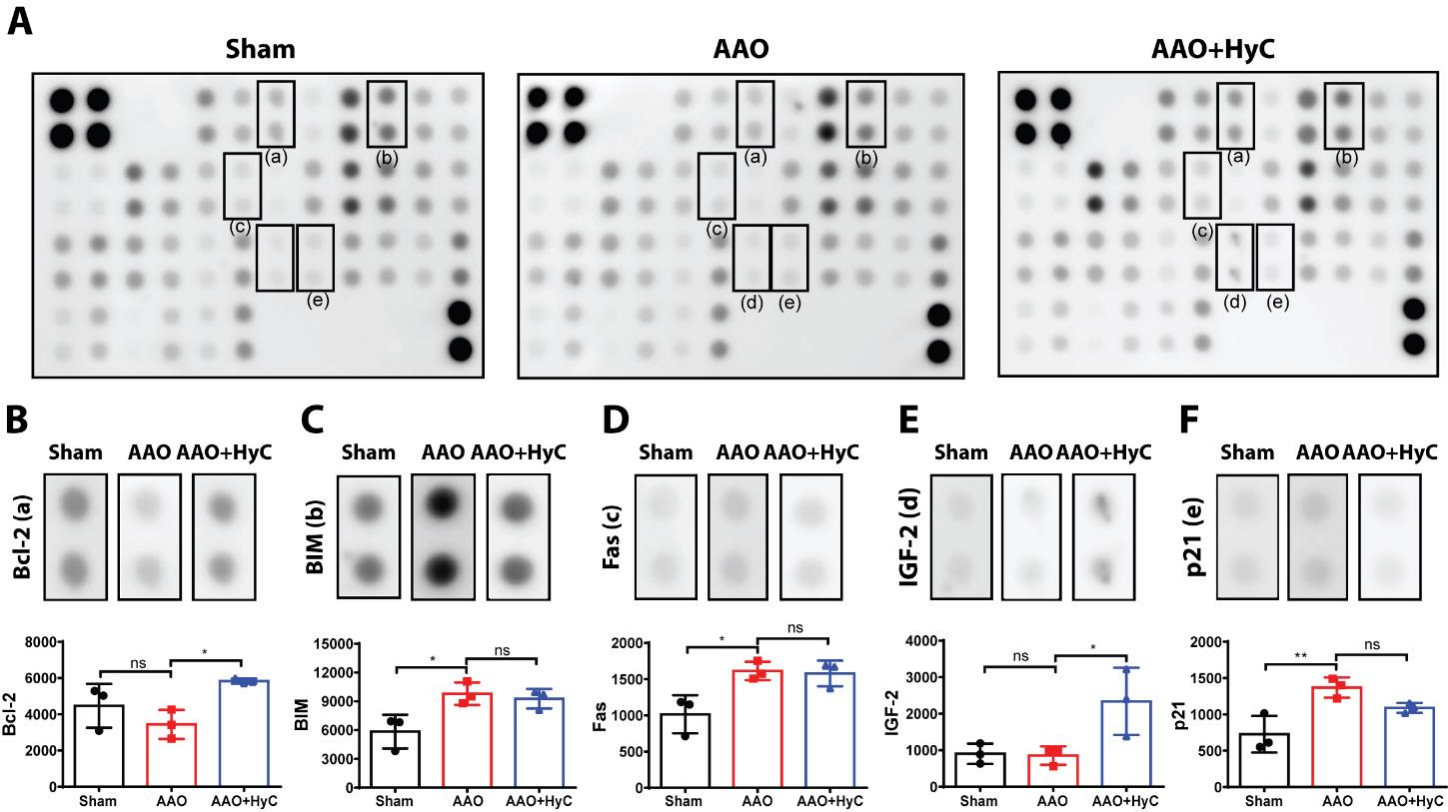
**Differential profile of apoptosis-related proteins in the brain and heart. (A)** Representative merged images of antibody microarrays for protein detection. **(B-F)** Bar graph showed the protein semi-quantification. After examining the 38 target proteins, five exhibited marked differences in expression among the three groups in the heart. *P<0.05, **P<0.01. ns, no significant difference. N=3 mice per group.

In the heart, AAO resulted in the upregulation of BCL2-like 11 (BIM), TNF receptor superfamily member 6 (Fas) and cyclin dependent kinase inhibitor 1A (p21) (Fig. 8C, D, F). HyC treatment did not inhibit the expression of the above proteins. However, the expressions of Bcl-2 and insulin-like growth factor 2 (IGF-2) in AAO+HyC group were significantly increased compared to AAO group (*P*<0.05) (Fig. 8B and E).

**Figure 9.**
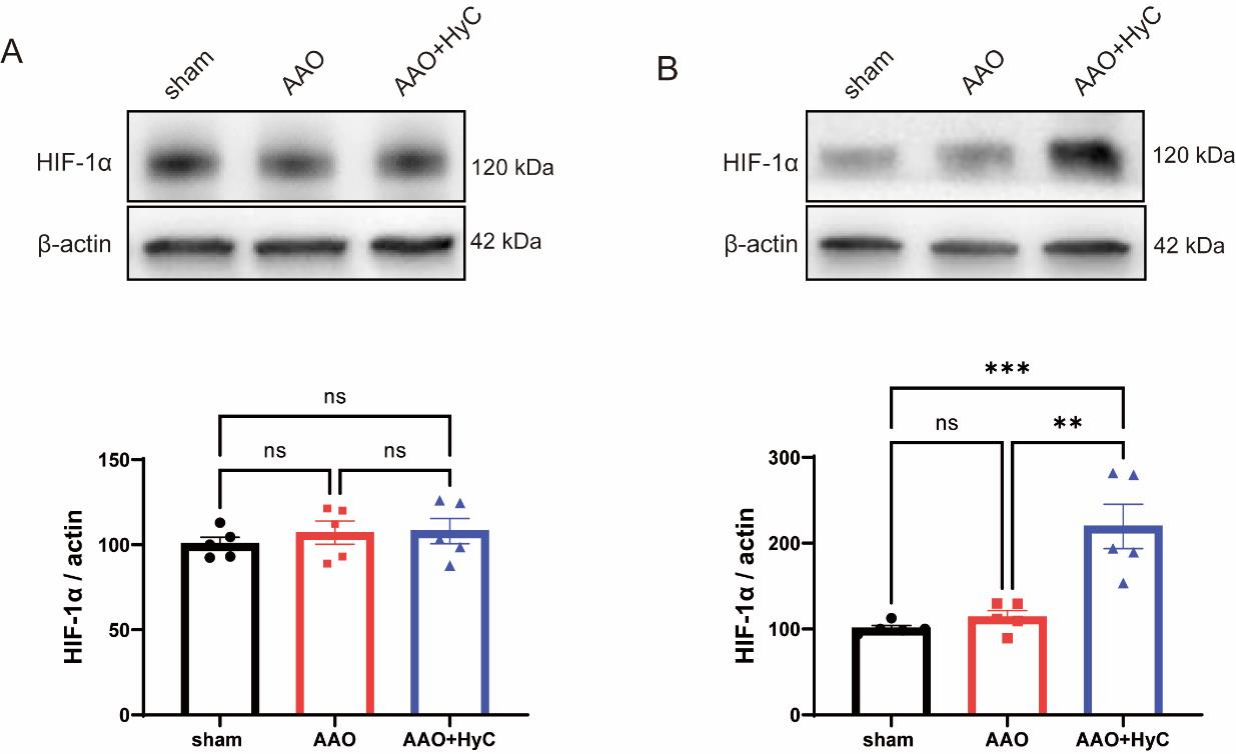
**HyC upregulated HIF-1α in the heart. (A)** Western blot analysis was conducted to assess the protein levels of HIF-1α in the brain of mice, followed by quantitative analysis. One-way ANOVA with Tukey’s honestly post hoc test was used. Each group, n=5, ns=no significant. **(B)** Western blot analysis was conducted to assess the protein levels of HIF-1α in heart of mice, followed by quantitative analysis. One-way ANOVA with Tukey’s honestly post hoc test was used. Each group, n=5, *** p<0.001, ** p<0.01.

As hypoxia-inducible factor 1 alpha (HIF-1α) is a crucial transcription factor that upregulates the expression of anti-apoptotic proteins and suppresses the expression of pro-apoptotic proteins during hypoxic conditions [18], we performed western blot analysis to quantify the levels of HIF-1α in the brain and heart. The results revealed no significant difference in the expression of HIF-1α between the sham group and the AAO group, or between the AAO+HyC group and the AAO group in the brain (Fig. 9A). However, HyC treatment upregulated the expression of HIF-1α in the heart (Fig. 9B), which corresponded to the expression of apoptosis-related proteins.

### HyC regulated inflammation-related factors in brain and heart

**Figure 10.**
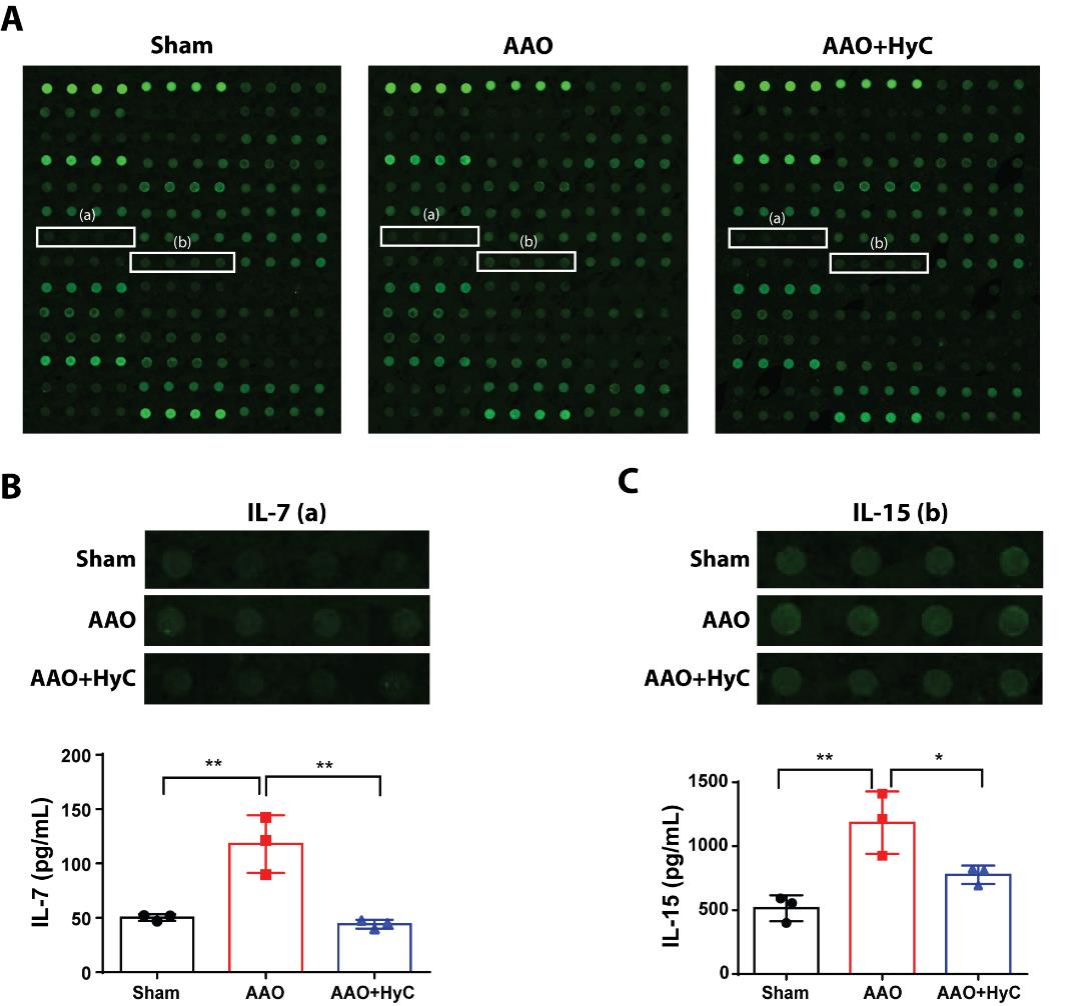
**Differential profile of inflammation-related proteins in the brain. (A)** Representative merged images of antibody microarrays for protein detection. **(B, D)** Bar graph showed the protein semi-quantification. After examining the 40 target proteins, two exhibited marked differences in expression among the three groups in the heart. The Kruskal-Wallis test with Dunn's *post hoc* test was used. *P<0.05, **P<0.01. ns, no significant difference. N=3 mice per group.

We compared the expression levels of inflammation-related proteins in the brain and heart among the sham, AAO, and AAO+HyC groups using an inflammation Antibody Array. We observed that out of the 40 target proteins in the brain, the expressions of interleukin-7 (IL-7) and interleukin-15 (IL-15) were increased in the AAO group compared to the sham group (P<0.05) and subsequently decreased in the AAO+HyC group compared to the AAO group (P<0.05) (Fig. 10).

**Figure 11.**
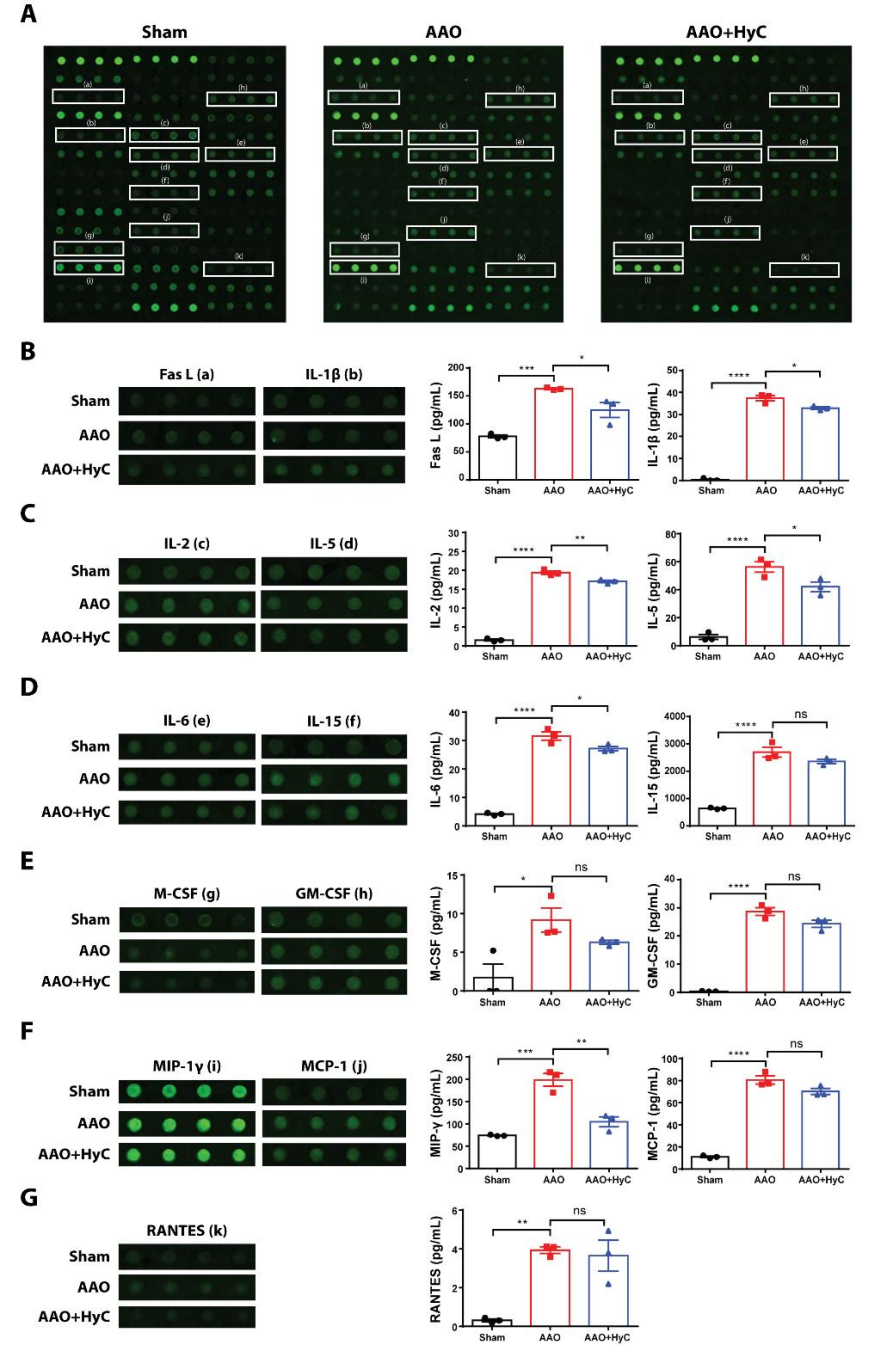
**Differential profile of inflammation-related proteins in the heart. (A)** Representative merged images of antibody microarrays for protein detection. **(B-G)** Bar graph showed the protein semi-quantification. After examining the 40 target proteins, eleven exhibited marked differences in expression among the three groups in the heart. The Kruskal-Wallis test with Dunn's *post hoc* test was used. *p<0.05, **p<0.01. ns, no significant difference. N=3 mice per group.

In the heart, eleven proteins exhibited marked differences in expression among the three groups (Fig. 10). The expressions of Fas ligand (FasL), interleukin-1β (IL-1β), interleukin-6 (IL-6), interleukin-2 (IL-2), interleukin-5 (IL-5), interleukin-15 (IL-15), colony-stimulating factor 1 (M-CSF), colony-stimulating factor 2 (GM-CSF), macrophage inflammatory protein 1γ (MIP-1γ), monocyte chemotactic protein-1 (MCP-1), and regulate and activate normal T-cell expression secretory factors (RANTES) were significantly increased in the AAO group compared to the sham group (P<0.05) (Fig. 11). However, treatment with HyC significantly reversed the expression of FasL, IL-1β, IL-2, IL-5, IL-6, MIP-1γ (Fig. 11).

## DISCUSSION

Ischemia-reperfusion is linked to elevated rates of morbidity, as well as increased incidences of myocardial infarction, stroke, and acute renal injury [19, 20]. There have been numerous studies on the ischemia-reperfusion injury resulting from the blockage of blood vessels supplying organs. However, the impact and molecular mechanism of injury to proximal and distal organs caused by abdominal aortic occlusion are not well understood. The present study contributed to our understanding of the impact of HyC strategy on directly (i.e. kidney, limb muscle) and remotely (i.e. heart, brain) affected organ. The current study revealed that HyC improved biochemiacal profile as reflected by improved kidney and heart function and reduced tissue destruction at a systemic level. And further protein antibody array of brain and heart tissue revealed that HyC regulates apoptosis and inflammatory factors.

Previous reports have demonstrated that AAO could result in abnormal kidney, liver and heat function [4, 21, 22]. Our study showed that renal function and cardiac function were significantly impaired in the AAO model, and HyC reversed the injury. In a meta-analysis, Wever et al analyzed 58 experimental animal studies and found that ischemic preconditioning resulted in reduced levels of creatinine, blood urea nitrogen, and histological damage compared to untreated animals [23]. Karageorgiadi et al reported that remote ischemic conditioning reduced both renal and myocardial injury. Our findings come in line with these results. It is worth noting that in our experiments, liver function markers TBIL and ALP did not show significant changes. This indicates that AAO and HyC treatment do not have a significant impact on liver function. Our result is inconsistent with Aydin's previous reporting [24]. The inconsistency between our results and Aydin's previous findings regarding the impact of AAO on liver function markers could be attributed to differences in experimental design, specific conditions or factors not accounted for, variations in timing and markers assessed, as well as inherent biological variability. Further studies are needed to elucidate the underlying mechanisms and clarify the discrepancies observed.

The results of our study provide important insights into the histopathological effects of AAO/reperfusion on proximal and distal organs. Our results demonstrate that AAO can induce pathological damage in the kidney, lower limb muscle, heart, and brain. These findings are consistent with previous studies that have reported AAO-induced tissue damage[4, 5, 25], but our study provides a more comprehensive understanding of the cellular and tissue-level changes that occur during AAO reperfusion. Our study also highlights the potential of HyC as an effective intervention for individuals at risk of pathological injury of organs following AAO. The fact that HyC was able to reverse the pathological changes induced by AAO is a significant finding that may have important clinical implications. Clinicians may need to consider interventions to promote HyC as a means of reversing the cellular and tissue-level changes that occur during AAO reperfusion. Our findings suggest that clinicians may need to monitor patients for potential kidney, low limb muscle, brain, and heart damage following AAO, and they should consider interventions to promote HyC as a means of reversing these changes.

Apoptosis is a highly regulated process of programmed cell death that plays a crucial role in normal development, tissue homeostasis, and elimination of damaged cells [26]. Dysregulation of apoptosis is associated with various pathological conditions, including neurodegenerative diseases and cardiovascular disorders [27]. The expression of several apoptosis-related proteins can modulate the apoptotic signaling pathway, and changes in their expression levels may contribute to the pathogenesis of these diseases [28]. Although our histopathological results showed no significant apoptosis in the heart and brain following AAO which is consistent with our previous study [29], other previous studies have demonstrated that this condition can cause cell death or apoptosis in proximal organs [25], [30], [31]. The brain and heart have the highest oxygen demand among all organs in the body, thus making it the most sensitive to hypoxia/ischemia [32], [33]. Therefore, we aim to investigate whether apoptosis-related factors have changed in these organs, and further clarify the regulatory effect of HyC on these proteins. By doing so, we hope to gain a better understanding of the molecular mechanisms underlying the effects of abdominal aortic ischemia-reperfusion on the heart and brain, and to clarify the protective mechanism of HyC.

In our study, we observed that AAO induced the upregulation of TNF-α and TRAIL-R2 in the brain. TNF-α is a pro-inflammatory cytokine that can induce apoptosis through the activation of caspases and the death receptor pathway [34]. TRAIL-R2 is a death receptor that can induce apoptosis upon binding to its ligand, TRAIL [35]. Therefore, these results suggested that AAO induced apoptosis may be induced through the death receptor pathway and the activation of caspases. However, treatment with HyC did not affect the expression of these proteins. On the other hand, HyC treatment significantly increased the expression of cIAP2, which is an anti-apoptotic protein that can inhibit caspase activation and promote cell survival [36]. This result suggests that HyC may inhibit apoptosis by upregulating the expression of anti-apoptotic proteins.

In the heart, AAO increased the levels of BIM, Fas, and p21. BIM induces apoptosis through caspase activation and the mitochondrial pathway [37], while Fas triggers apoptosis when bound to FasL.L [35]. P21, on the other hand, causes cell cycle arrest and apoptosis. [38]. These findings indicate that AAO induces apoptosis in the heart through multiple pathways. However, HyC treatment did not inhibit the expression of these proteins. Instead, it significantly increased the expression of Bcl-2 and IGF-2, which are anti-apoptotic proteins that can promote cell survival and inhibit apoptosis [39]. These findings suggest that HyC may regulate the differential expression of apoptosis-related proteins in a tissue-specific manner.

Previous studies have shown that HyC can regulate the differential expression of various proteins involved in apoptosis [18]. For example, hypoxia-inducible factor 1 alpha (HIF-1α) is a transcription factor that is upregulated under hypoxic conditions and can induce the expression of various anti-apoptotic proteins, such as Bcl-2 and Bcl-xL [40]. HIF-1α can also inhibit the expression of pro-apoptotic proteins, such as Bax and Bak [10]. Our results showed that HyC treatment upregulated the expression of HIF-1α in the heart, which corresponded to the expression of apoptosis-related proteins. Mounting evidence has shown that inflammation play a critical role in systemic complications induced by AAO [21, 41]. Since the brain and heart has the highest oxygen demand among any organ, it is extremely vulnerable to ischemia or hypoxia. In both clinical and animal models of AAO, various pro-inflammatory cytokines like have been associated with tissue injury [42, 43].

In our study, we used an inflammation Antibody Array to compare the expression levels of inflammation-related proteins in the brain and heart among the sham, AAO, and AAO+HyC groups. The results showed that AAO induced a significant increase in the expression of IL-7 and IL-15 in the brain. IL-7 and IL-15 are cytokines that play a crucial role in the regulation of immune responses and inflammation [44]. They are produced by various cell types, including T cells, B cells, and macrophages, and can promote the proliferation and activation of immune cells [44]. Therefore, the upregulation of IL-7 and IL-15 in the brain following AAO suggests that AAO may induce an inflammatory response in the brain through the activation of immune cells.

In the heart, AAO induced a significant increase in the expression of FasL, IL-1β, IL-2, IL-5, IL-6, MIP-1γ, MCP-1, GM-CSF, M-CSF, and RANTES. FasL is a cytokine that can induce apoptosis through the activation of Fas [45]. IL-1β and IL-6 are pro-inflammatory cytokines that can induce the production of other pro-inflammatory cytokines and chemokines [46]. IL-2 and IL-5 are cytokines that can promote the proliferation and activation of immune cells [47, 48]. MIP-1γ, MCP-1, GM-CSF, M-CSF, and RANTES are chemokines that can recruit immune cells to the site of inflammation [49, 50]. Therefore, the upregulation of these proteins in the heart following AAO suggests that AAO may induce an inflammatory response in the heart through the activation of immune cells and the production of pro-inflammatory cytokines and chemokines. However, treatment with HyC significantly reversed the expression of FasL, IL-1β, IL-2, IL-5, IL-6, and MIP-1γ in the heart. This suggests that HyC may inhibit the AAO-induced inflammatory response in the heart by downregulating the expression of pro-inflammatory cytokines and chemokines. Further studies are needed to determine the molecular mechanisms underlying the anti-inflammatory effects of HyC and to explore the potential therapeutic applications of HyC in the prevention and treatment of AAO-induced organ damage.

One limitation of our study is that it was conducted in an animal model, and it is unclear whether the findings can be extrapolated to humans. Future studies should investigate the effect of HyC on AAO/reperfusion injury in human subjects to determine the clinical relevance of our findings. Another limitation is that our study did not extensively investigate the long-term effects or potential adverse effects of HyC. Therefore, future research should include long-term follow-up studies in both animal models and human subjects to assess the safety profile and potential risks associated with HyC treatment.

Overall, the results of this study suggest that HyC treatment may be an effective perioperative strategy to reduce the pathological damage caused by AAO on various organs. Furthermore, our study provides important insights into the potential molecular mechanisms underlying the effects of HyC on AAO reperfusion-induced tissue damage. Specifically, our results suggest that HyC may regulate apoptosis-related proteins in the brain and heart, as well as inflammation-related factors in these organs. Overall, our study highlights the potential of HyC as a promising therapeutic strategy for mitigating tissue damage induced by AAO reperfusion.
